# On the *SHELX*, *PLATON* and IUCr/*checkCIF* crystal structure validation platform

**DOI:** 10.1107/S2053229626009319

**Published:** 2026-09-23

**Authors:** Anthony L. Spek

**Affiliations:** a Utrecht University, Utrecht, The Netherlands

**Keywords:** *SHELX*, *PLATON*, *checkCIF*, validation

## Abstract

With *checkCIF* the IUCr has created a unique platform for the validation and archiving of the result of a small-mol­ecule crystal structure study. That information, supplied in CIF (crystallographic information file) format, should include not only the numerical results of that study but also much of the supporting experimental data and details of the procedures used during the structural investigation. This not only allows for a proper validation of a structure report but also provides an option for a future re-evaluation or use of the data for an unrelated study. The experimental data might be difficult to obtain again or even unique. George Sheldrick’s *SHELX* software was an early adopter of CIF and with that has played a major role in the success of the IUCr validation effort. IUCr/*checkCIF* initially started out with tests that check for the completeness of the supplied data, along with tests that check whether their values are within the expected range. Currently, the *PLATON* software carries out most of the tests in the IUCr/*checkCIF* software framework. Validation issues are reported as a set of ALERTS along with a short clarification. All ALERT messages should be inspected, addressed with a correction where applicable or a short explanation of a special situation. Some ALERT messages are informative but do not necessarily need a comment. More detailed information about a reported issue, such as warnings about missed twinning, missed additional symmetry or voids, might be obtained with a locally installed version of the *PLATON* software using the same tools on which those ALERTS are based. As an example of this, the use of the *PLATON*/*TwinRotMat* tool for the detection of twinning is discussed along with some details about the underlying algorithm. Three twinning examples are presented, covering merohedral, pseudomerohedral and non-merohedral twins, to illustrate the additional twinning information that can be obtained beyond what is shown as an ALERT in the IUCr/*checkCIF* report.

## Abstract 2

With *checkCIF* the IUCr has created a unique platform for the validation and archiving of the result of a small-mol­ecule crystal structure study.

## Introduction and history

*SHELX* (Sheldrick, 2008 ▸; Usón & Herbst-Irmer, 2025 ▸) was written in the 1970s as a rather complete software package for the X-ray crystal structure determination and refinement of small-mol­ecule structures. It was initially distributed as *SHELX-76* by George Sheldrick out of Cambridge University (UK) in the form of a box with 2000 punch cards. It included the Fortran source, an extensive user manual and test examples, all in compressed file format, along with a small Fortran program to decompress the encoded files. Later versions were distributed on magnetic tape. It was easily implementable on the Utrecht University CDC6400 mainframe computer and could, due to its small memory footprint, even be used to run small jobs in timesharing terminal mode, thus avoiding issues associated with the less convenient and slow turnaround batch mode processing. This author even managed to implement *SHELX-76*, with some adaptations, including a primitive virtual memory system to handle a larger than standard full-matrix least-squares refinement, on our in-house 32 kB Data General Eclipse minicomputer equipped with a 5 MB removable disk. That was possible only due to the excellent program structure of *SHELX-76* that allowed for code overlay of subroutines. The distributed version of *SHELX-76* was stable and complete for a long time and was never updated. The structure solution functionality was separated into a new and more powerful program, *SHELXS* (Sheldrick, 2008 ▸), later to be superseded by *SHELXT* (Sheldrick, 2015*a* ▸). The structure refinement functionality was replaced by the completely new *SHELXL* program (Sheldrick, 2015*b* ▸) that now includes facilities for twin refinement.

*PLATON* (Spek, 2003 ▸; Spek, 2009 ▸; Spek, 2018 ▸; Spek, 2020 ▸) originated in the 1980s as a collection of software tools for the analysis and reporting of the results of a single-crystal structure determination performed with *SHELX-76*. It was developed in the context of the National Crystal Structure Service Facility in Utrecht, the Netherlands. It included, apart from various geometry calculations, tools such as *ADDSYM* for the detection of missed symmetry and later a preliminary version of the *VOID* and *SQUEEZE* (Spek, 2015 ▸) tools for the detection and handling of voids in the structure containing disordered solvents as part of a structure refinement. Distribution of *PLATON* was done at that time electronically *via* DECNET, a predecessor of the Inter­net.

IUCr/*checkCIF* as a project started in the 1990s to automate structure validation of the exploding number of structures reported in the IUCr journals, particularly in the new *Acta Crystallographica Section E* journal intended for short structural reports (Strickland *et al.*, 2006 ▸). The idea was to archive good structure reports that, though valuable as a Cambridge Structural Database (CSD; Groom *et al.*, 2016 ▸) entry, would not make it into a full article. A tool was needed to manage the refereeing process. The determination of crystal structures had become increasingly routine owing to advancements in diffraction hardware, specifically the shift from slow one-dimensional detectors to significantly faster two-dimensional detectors, improving data collection speed by at least a factor of ten. Direct methods, such as *MULTAN* (Germain *et al.*, 1971 ▸), *SHELXS* (Sheldrick, 1990 ▸), *SIR* (Burla *et al.*, 1989 ▸) and *DIRDIF* (Beurskens *et al.*, 1996 ▸), had made structure determination more accessible for non-specialists engaged in synthetic chemistry. The latter applies even more today with the now available *SHELXT* (Sheldrick, 2015*a* ▸) structure solution software.

Multiple other general-purpose crystallographic software packages used today have their origin in the 1970s as well [*CRYSTALS* (Betteridge *et al.*, 2003 ▸), *JANA* (Petricek & Dusek, 2000 ▸), *WinGX* (Farrugia, 2012 ▸), *etc*.]. They were running on a variety of and often incompatible university mainframe hardware. At that time computers came with a large variety of machine word lengths such as 12, 16, 24, 27, 36, 60 or 64 bits as opposed to the current 8-bit byte standard, which made cross-implementation of software a major issue. Another major issue was the number of characters packed in a machine word and their encoding. The first version of *SHELX*, made available as *SHELX-76*, turned out to be probably the easiest one to implement on the variety of mainframe hardware platforms (IBM, CDC, DEC, UNIVAC, *etc*.) and even on departmental mini-computers (*e.g.* Data General Nova and Eclipse and Digital Equipment VAXs). Current *SHELX* software versions are conveniently made available as executables only for the Windows, LINUX and MacOS platforms. That made *SHELXL* software, until recently, the most cited standalone refinement tool. *SHELXL* is still the most used refinement tool running behind the Graphical User Inter­face (GUI) of current crystallographic software packages, such as *OLEX2* (Dolomanov *et al.*, 2009 ▸), *ShelXle* (Hübschle *et al.*, 2011 ▸), *WinGX* (Farrugia, 2012 ▸) and commercial packages that come with the diffractometer hardware.

The exchange of structural data was complicated before the 1990s outside the software and hardware ecosystem used. *SHELX-76* uses a simple computer and human-readable free format .INS, .RES and .HKL file model for data input and output. Those files often served for data exchange and archival purposes. However, they lacked essential information such as s.u. values on the refined structural parameter values.

The results of a crystal structure study would be reported as atomic coordinate tables in a printed manuscript from which the content had to be retyped in computer-readable format for database entry or follow-up use. There were early attempts to create a data exchange standard (Brown, 1983 ▸). The one that survived to this day is the CIF standard (Hall *et al.*, 1991 ▸). CIF in its original design is a flexible free format using, often looped, datanames with the associated value system. It was first implemented in the multi-author *XTAL* crystallographic software system (Hall *et al.*, 1992 ▸) that has been depricated.

It was Syd Hall, co-author of the original CIF standard, at that time also Section Editor of *Acta Crystallographica Section C* and co-developer of the *XTAL* system, who convinced George Sheldrick to implement CIF for the archiving of the refinement results in the next release of the *SHELX* refinement software. That new version was *SHELXL-93* (Sheldrick, 2015*b* ▸). With that, the use of CIF rose steeply along with the growing popularity of *SHELXL*-based structure refinement. Other major software packages eventually complied with the CIF format as well.

CIF was adopted as the standard for data deposition and archiving for crystal structure publications in the IUCr journals. This was later extended as the format for submitting a structure report for *Acta Crystallographica Section E* and *Section C* articles. A logical next step was the development of the IUCr/*checkCIF* software aiming at the automatic creation of a validation report to be used as part of the refereeing process. The original IUCr/*checkCIF* software included mainly tests for the completeness of the supplied data and tests of their values being within acceptable ranges. Soon after, on invitation by Syd Hall, additional tests were included in IUCr/*checkCIF* as provided by the *PLATON* software, involving calculations to independently investigate difference electron-density maps, missed additional symmetry, missed twinning and voids with unaccounted residual electron density. The current IUCr/*checkCIF* implementation is a merger of the original IUCr/*checkCIF* and *PLATON*/*checkCIF* sets of tests and ALERTS. The *PLATON* generated ALERTS are referred to as PLATxxx in the IUCR/*checkCIF* report, where xxx is an ALERT number. Fig. 1 ▸ shows as an example the *PLATON*/*checkCIF* report for the merohedrally twinned CIJVEA structure (Kollitz *et al.*, 2023 ▸) to be discussed below in Section 3.2.

Traditionally, a crystal structure report was expected to include the supply and archiving of a printed *F*_o_/*F*_c_ table that supposedly allowed for detailed manual inspection of *F*_o_*versus F*_c_ consistency and data set completeness. Actual use of the observed data for follow-up calculations would however require laborious retyping and was thus rarely done. Retyping was sometimes done by Dick Marsh (Marsh & Spek, 2001 ▸) as part of his famous reports on missed higher symmetry in the literature. Accessibility of those reflection data improved when programs like *SHELXL* not only created a CIF file with the structural parameter data, but also a CIF-style structured FCF file containing the *F*_o_^2^, *F*_c_^2^ and σ(*F*_o_^2^) reflection data used in the final least-squares refinement. Such a file is not only useful for a more detailed analysis of the refinement results, but also useful for the detection of problems such as missed twinning and erroneous space-group assignments.

The availability of archived FCF files turned out to be key in the detection of a large set of unfortunately fraudulent structure reports, mainly published in *Acta Crystallographica Section E* (Harrison *et al.*, 2010 ▸). The deposition of an FCF file is now mandatory for the IUCr *Acta Crystallographica* journals as for other major journals. It was soon realized that the deposition of unmerged reflection data was even more relevant along with the instruction file used for the least-squares refinement. This is, in the case of a *SHELXL*-based refinement, implemented by embedding the .INS and .HKL files as a text value between two semicolons with associated datanames such as _shelx_res_file and _shelx_hkl_file in the CIF file. Simple extraction of those files from such a CIF, which can be done with the *PLATON* tool cif2shelx, makes reconstruction or improvement of a refinement easy. George Sheldrick himself found this concept particularly useful for testing his programs on archived data since the *SHELX* software was not designed to accept CIF files as input. Alternatively, refinement programs such as *OLEX2*/*Refine* (Dolomanov *et al.*, 2009 ▸) include the unmerged reflection data as a CIF-style loop in the .CIF file.

*CheckCIF* ALERTS come in four levels: A, B, C and G. The first three concern issues that generally should be investigated, corrected and/or commented on. G level ALERTS are often informative but may in combination also point at more serious issues to be addressed. *CheckCIF* ALERTS can be terse. The same may be the case with their associated short explanatory texts. Some ALERTS are based on *PLATON* tools, such as *ADDSYM* for missed symmetry and *TwinRotMat* for missed twinning. Those tools can be used for a more detailed analysis of the reported issues with a locally installed *PLATON* version. One of those tools, *TwinRotMat*, is discussed in Section 3. Details are presented of its algorithm and examples are given of its use as a tool to detect, investigate and handle twinning in the final refinement.

The Cambridge Crystallographic Data Center (CCDC) started the CSD database in the 1970s. In the early days, structure data were digitized from the published paper. Coordinates were tested against the reported bond and angle data. Inconsistencies, often due to typos, were corrected and missing data tracked from the authors. That laborious work has now been largely superseded with CIF-based data deposition and IUCr/*checkCIF* validation.

## *PLATON* and *checkCIF*

*PLATON* is a set of tools collected in a single program. Most of those tools can be invoked individually (Fig. 2 ▸) or run in a programmed sequence. An example is the *Calc ALL* command that automatically executes, given CIF and FCF files as input, most of the available *PLATON* geometry and analysis tools. A similar run is done by invoking the *VALIDATION* tool where detected issues are temporarily written to an inter­mediate file. Subsequently, those issues are sorted, analyzed and reported as ALERTS with associated levels of importance. When no structure factor FCF file is present, the *PLATON* software launches an external *SHELXL* job to reconstruct the final refinement result, based on the CIF-embedded final .INS and .HKL files, and to analyze it.

*PLATON* uses its own built-in CIF reader to accommodate the variety of CIFs created by non-*SHELXL* refinement programs. Several of them use datanames of their own design to report features specific to that program. Unfortunately, not all relevant information about a crystal structure determination may be available as the value of an official CIF dataname or presented as such by the CIF-generating refinement software. With *SHELXL*, additional information can be extracted from the embedded .INS file. As an example, the proper reporting of twinning in the CSD is often an issue. One aspect of this is that many structures refined as inversion twins are marked ‘twinned’ in the CSD. This is obviously correct but not very helpful when you are inter­ested in cases of non-inversion twinning. With a *SHELXL*-based refinement, that type of information can be gleaned from the presence of BASF, TWIN or HKLF 5 instructions in the embedded .INS file. Other refinement software may include a CIF-style loop containing the twin domain information, though without explicit information about the type of twinning. Sometimes it is not even clear whether a correction for twinning was performed as part of the final refinement. *PLATON*/*checkCIF* will analyze the FCF data for non-inversion-twinning and report on it independently of whether they were corrected for twinning or not. Similar considerations apply for the reporting of the value of the Flack parameter in its various incarnations (Flack *et al.*, 1983 ▸; Parsons *et al.*, 2013 ▸; Hooft *et al.*, 2008 ▸; Hooft *et al.*, 2009 ▸; Hooft *et al.*, 2010 ▸)

## Twinning detection

Preferably, diffraction data are collected on a single crystal rather than on a twinned specimen; see Parsons (2003 ▸) for an introduction to twinning. Structure determination is more straightforward with single-crystal diffraction data. The quality of the result with single-crystal data is often more satisfactory. Twinning needs to be detected, a twinning model needs to be found and subsequently included in the refinement model. Twinning can be missed during data collection resulting in problems with the structure solution and/or refinement. Sometimes only twinned crystals are available for data collection. Inspection of the selected crystal with a polarizing microscope might avoid this problem. Preliminary structure solution with standard tools such as *SHELXT* (Sheldrick, 2015*a* ▸) may still be possible when the twinning is minor and difficult when the correct space group is unclear. Subsequent structure refinement will be unsatisfactory, with high *R*1 values and spurious unexplained electron-density peaks in difference Fourier maps when the twinning is not included in the refinement model. Fig. 3 ▸ gives an example of major residual density peaks when twinning is not handled properly (see Section 3.2). Refinement programs such as *SHELXL* can account for twinning once a twinning model is available. Such a twinning model can sometimes be derived from an inspection of the diffraction images for diffraction spots not fitting in the structure model lattice used, *i.e.* belonging to a twin-related lattice. That is not always the case. Crystals twinned by merohedry (Nespolo *et al.*, 2014 ▸) have an exact overlap of lattices related by a symmetry operation of the lattice that is not a symmetry element of the structure.

*PLATON* includes a tool, *TwinRotMat*, that aims to provide the relevant twinning information to be included in the final least-squares refinement. It is based on an analysis of the FCF data of a (preliminary) *SHELXL*-based refinement. The development of the *TwinRotMat* algorithm was inspired by a report of an alternative algorithm, *ROTAX*, by Parsons & Gould (2001 ▸), that also uses the *F*_o_^2^ >> *F*_c_^2^ outliers in an FCF file, though in a very different way, to obtain a proposed twinning matrix [see Cooper *et al.* (2002 ▸) for its implementation in *CRYSTALS*].

*SHELXL*, in its general form, takes twinning into account in the least-squares refinement by using a reflection file (type HKLF 5) where for each primary reflection the indices of overlapping reflections are specified. This may address both cases of partial and complete diffraction lattice overlap. The associated twinning domain fraction(s) is/are refined. The special case of complete overlap can also be more conveniently handled with the TWIN/BASF instruction set specifying one twinning matrix and the twinning fraction(s).

### *TwinRotMat* algorithm

Reflection intensities of a twinned crystal may include an additional contribution of an overlapping twin-related reflection. The affected reflections manifest themselves in a preliminary refinement as a significant number of outliers with *F*_o_^2^ >> *F*_c_^2^. The *PLATON*/*TwinRotMat* tool uses those outliers for the construction of a twinning model. The central idea behind *TwinRotMat* is to make use of the fact that overlapping reflections must have the same diffraction angle theta (θ). Adding up the two diffraction vectors **h** and **h′** of the reflections involved will lead to a reciprocal lattice vector that, when reduced by taking out a common factor, will suggest a candidate for a twofold twin rotation axis. The associated twinning matrix can be derived with that information and used for a final *SHELXL* refinement. There can be a number of **h′** reflections satisfying within a tolerance value the theta criterium for a given outlier reflection **h**, with associated rotation axes. The correct rotation axis is expected to show up in a statistical analysis of all such proposals for a set of outlier reflections. By default, 50 reflections, **h**, with the largest *F*_o_^2^ >> *F*_c_^2^ values are selected. The result is a list of potential twin rotation vectors with their frequency of occurrence. That list is sorted on the frequency of occurrence. Subsequently, an estimated twinning factor (BASF for *SHELXL*) is calculated by a least-squares fitting of *F*_o_^2^(**h**) with *F*_c_^2^(**h**)_twin_ values for each of the more frequently occurring twin axis candidates, where *F*_c_^2^(**h**)_twin_ = (1.0 − BASF)*F_c_^2^(**h**) + BASF*F_c_^2^(**h′**). Finally, up to four twin matrices, ordered on their effect on lowering the *R*1 value, are listed.

A low index direct lattice vector, **v**, with an angle closest to the reciprocal lattice vector **h** is determined as well, along with its associated rotation matrix and its *R*1 lowering effect. Depending on the largest *R*1 lowering effect, the rotation matrix for either **h** or **v** is shown. The angle between the reciprocal lattice vector **h** and direct lattice vector **v** will be zero for twofold axes consistent with the lattice symmetry.

Three types of twinning can be handled with *TwinRotMat*: merohedral, pseudomerohedral (see also Guzei *et al.*, 2012 ▸) and non-merohedral, as discussed below.

### Merohedral twinning example

Fig. 4 ▸ shows a published structure (Kollitz *et al.*, 2023 ▸) with CSD refcode CIJVEA, crystalizing in the space group *P*
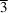
*c*1, where the crystal lattice has hexa­gonal symmetry and the structure only trigonal symmetry. The projection down the *c* axis of the content of the unit cell shows overlapping mol­ecules related by the *c*-glide of the space group. Fig. 5 ▸ shows the result of a *TwinRotMat* calculation, indicating in green that the structure is twinned with a twofold rotation axis about the direct lattice vector [001] with a BASF of 0.45. A lowering of the *R* value by −0.10 is predicted when applied in the final least-squares refinement. This result is consistent with the published twin refinement model. The next highest proposed twinning axis appears to have nearly no *R*1 value lowering effect and can be rejected. Final refinement with *SHELXL* can proceed with either a HKLF 5 file created by *PLATON*/*TwinRotMat* or with *SHELXL* instructions:

TWIN −1 0 0 0 −1 0 0 0 1 2

BASF 0.45

Fig. 6 ▸ displays a section of the reciprocal lattice for the *l* = 1 zone, showing the completely overlapping lattices for the twofold rotation about [001]. Reflections with *F*_o_^2^ >> *F*_c_^2^ are marked with green circles.

### Pseudomerohedral example

Fig. 7 ▸ shows the *TwinRotMat* result for a published structure (Langer *et al.*, 2004 ▸) with CSD refcode WUXXEU. It is reported in the ortho­rhom­bic space group *Cmcm*, pseudomerohedrally twinned by rotation about a threefold axis [001]. It was refined with the *SHELXL* instructions:

TWIN −0.5 0.5 0 −1.5 −0.5 0 0 0 1 3

BASF 0.21 0.21

*TwinRotMat* reports two twofold axes, [110] and [1
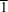
0], perpendicular to the authors threefold axis [001]. The angle between the two is 120°. Fig. 8 ▸ illustrates the respective two twofold rotation twin operations in the *l* = 1 reciprocal lattice zone. Least-squares refinement based on a HKLF 5 file created with *TwinRotMat* using the two proposed twinning matrices resulted in a refinement comparable with the published one with the same 0.58 0.21 0.21 twin volume fractions.

### Non-merohedral example

Fig. 9 ▸ shows the *TwinRotMat* result for a published structure (Nirmal Ram *et al.*, 2022 ▸) with the CSD refcode LUMKIP01 in the space group *P*2_1_/*c*. A twofold twinning operation about a* is detected with a predicted volume ratio of 56:44 that the authors refined to 0.52:0.48 with a *SHELXL*-type HKLF 5 reflection file least-squares refinement. Fig. 10 ▸ illustrates the non-merohedral twinning overlap in the *k* = 0 reciprocal lattice zone.

## Concluding remarks

As detailed above, *PLATON*/*checkCIF* was developed within the *SHELX* environment. Other refinement software pack­ages generally generate CIFs that follow the *SHELXL* and IUCr-required CIF model as close as possible. *PLATON*/*checkCIF* attempts to accommodate their differences, such as unofficial datanames, as much as possible. A major difference with non-*SHELXL*-created CIFs is that it is in most cases not possible to reconstruct the reported refinement exactly. Access to the associated refinement software will be necessary. To evaluate the refinement in such a case, an externally supplied FCF associated with the CIF will be required.

Currently, *checkCIF* mainly addresses the validation of mainstream small-mol­ecule structure reports. CIFs reporting structures such as those based on powder data, using non-spherical scattering factors or incommensurate structures are only partially validated. They will need the creation and inclusion of alternative validation tools to the IUCr/*checkCIF* platform.

*PLATON*/*checkCIF* and IUCr/*checkCIF* are not completely identical. The latter contains tests that are not available in *PLATON*/*checkCIF* and *vice versa*. IUCr/*checkCIF* is man­aged by the IUCr editorial staff in Chester (UK).

The earlier *SHELX* versions were distributed in source code without dependence on external libraries. They still compile happily on current hardware. The current version of *SHELX* software is supplied as executable and depends on libraries, mainly to speed up the calculations, for Intel-based hardware which might become an issue to be solved in the future. *PLATON* is distributed in source code but depends on the graphics libraries used (X-Windows).

The majority of current *SHELX* and *PLATON* users now use that software behind the GUI of *OLEX2* or commercial packages bundled with the diffractometer hardware.

## Software availability

The *PLATON* Fortran source code is available, free-of-charge, from https://platon.nl/xraysoft. A small C language routine is included to handle the GUI graphical inter­face with the X-Window system. It needs to be compiled and runs natively on UNIX-like platforms such as MacOS, Linux and WSL on Microsoft Windows. A stripped *PLATON* version without GUI graphics is available for the *checkCIF* validation functionality only. A compiled version for the Microsoft Windows platform is available from Dr Louis Farrugia at https://www.chem.gla.ac.uk/~louis/software/platon.

## Figures and Tables

**Figure 1:**
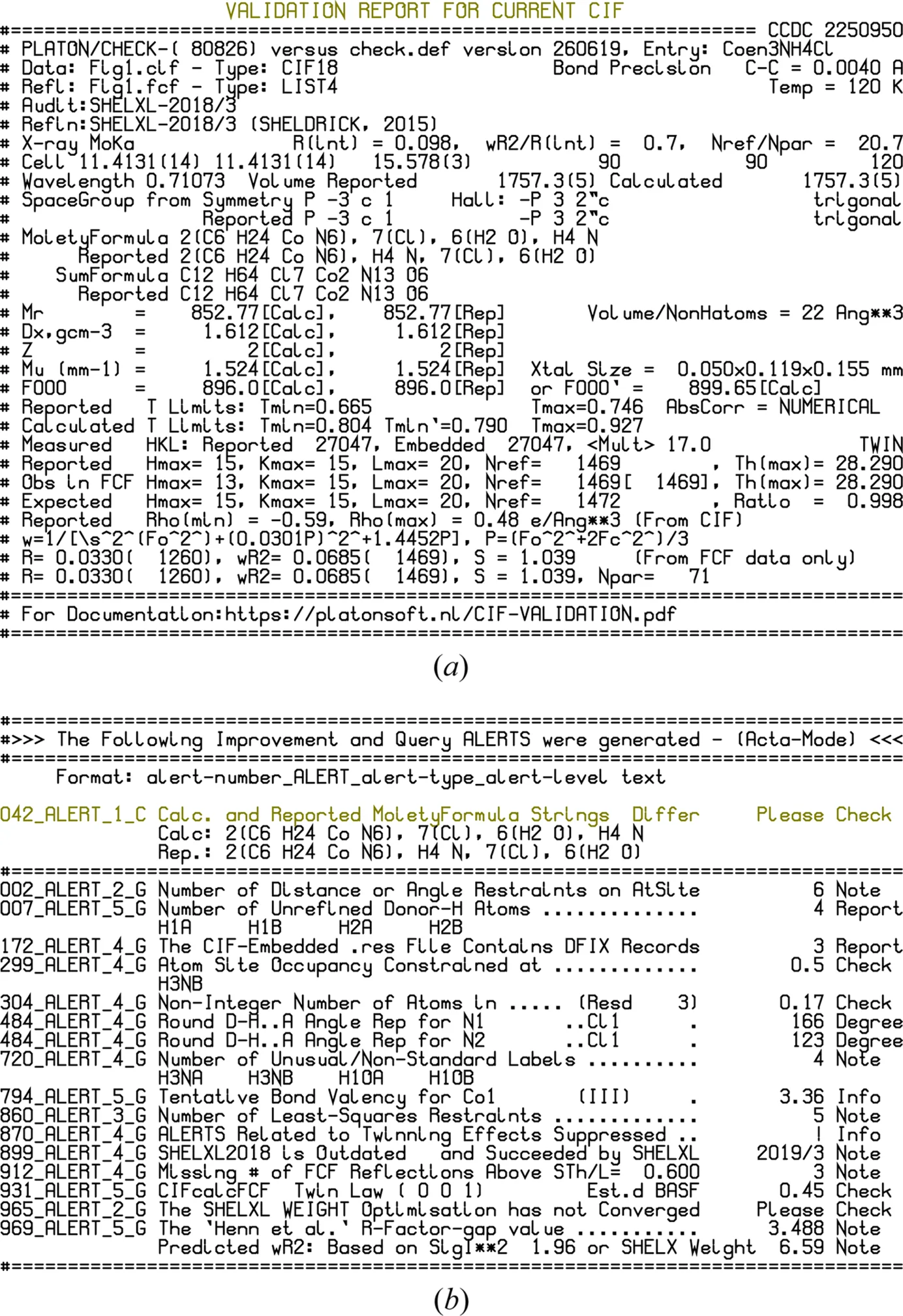
*PLATON*/*checkCIF* report for CIJVEA. (*a*) The relevant numerical information is shown. Both reported and calculated values are presented as derived from the data in the CIF and FCF files. (*b*) The mainly informative G level ALERTS are shown. ALERT #931 reports on a proposed twinning law that may or may not have been taken care of in the least-squares refinement.

**Figure 2:**
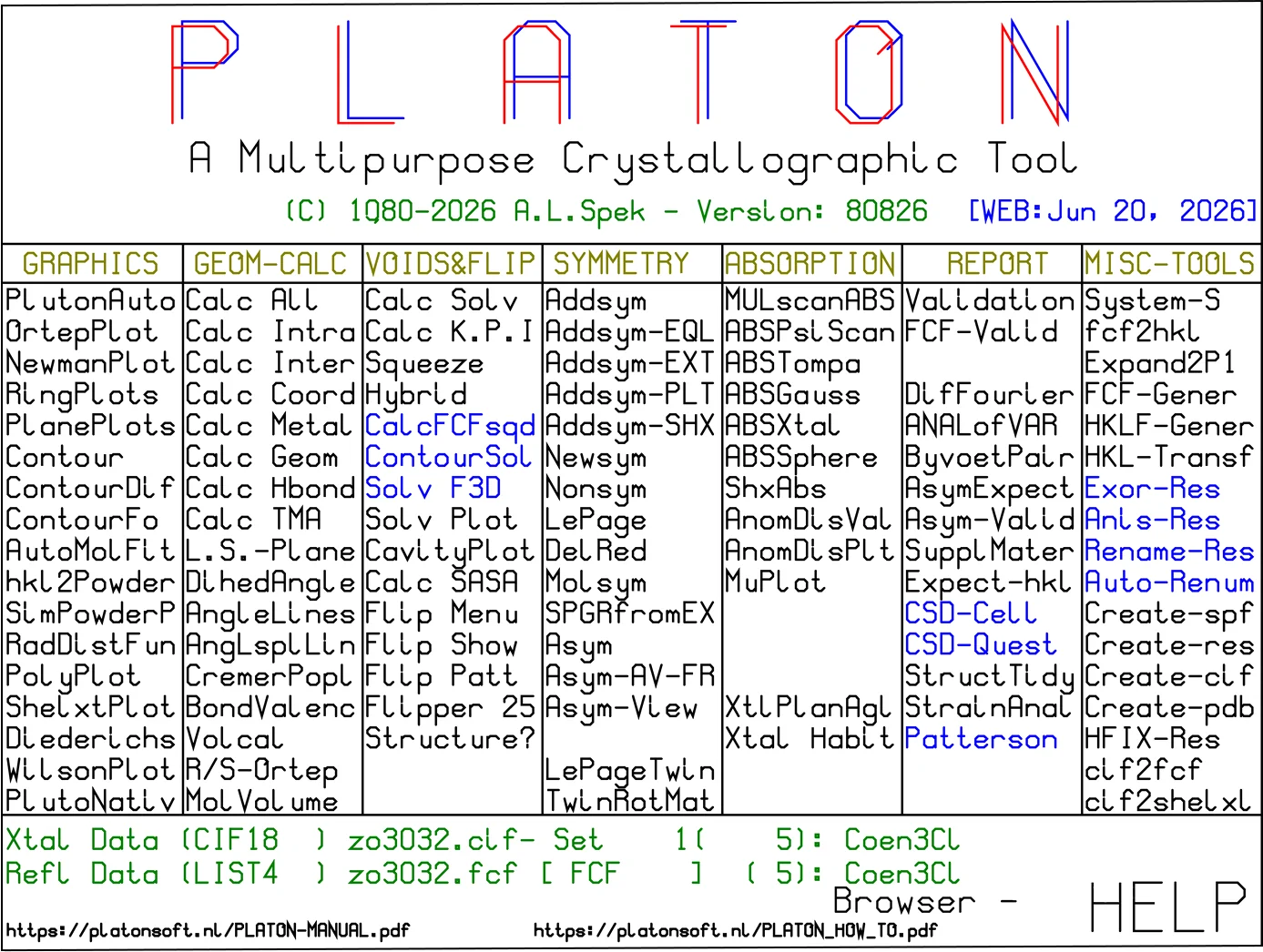
Opening display of the *PLATON* software showing the available clickable tools and options such as *Validation* and *TwinRotMat*. Both tools require a CIF and associated FCF file for the calculations. *PLATON* will automatically recreate the final FCF with a spawned *SHELXL* job, when not found, using the embedded data in the supplied *SHELXL*-created CIF. The additional option window on the right and optional keyboard data entry window at the bottom are not shown.

**Figure 3:**
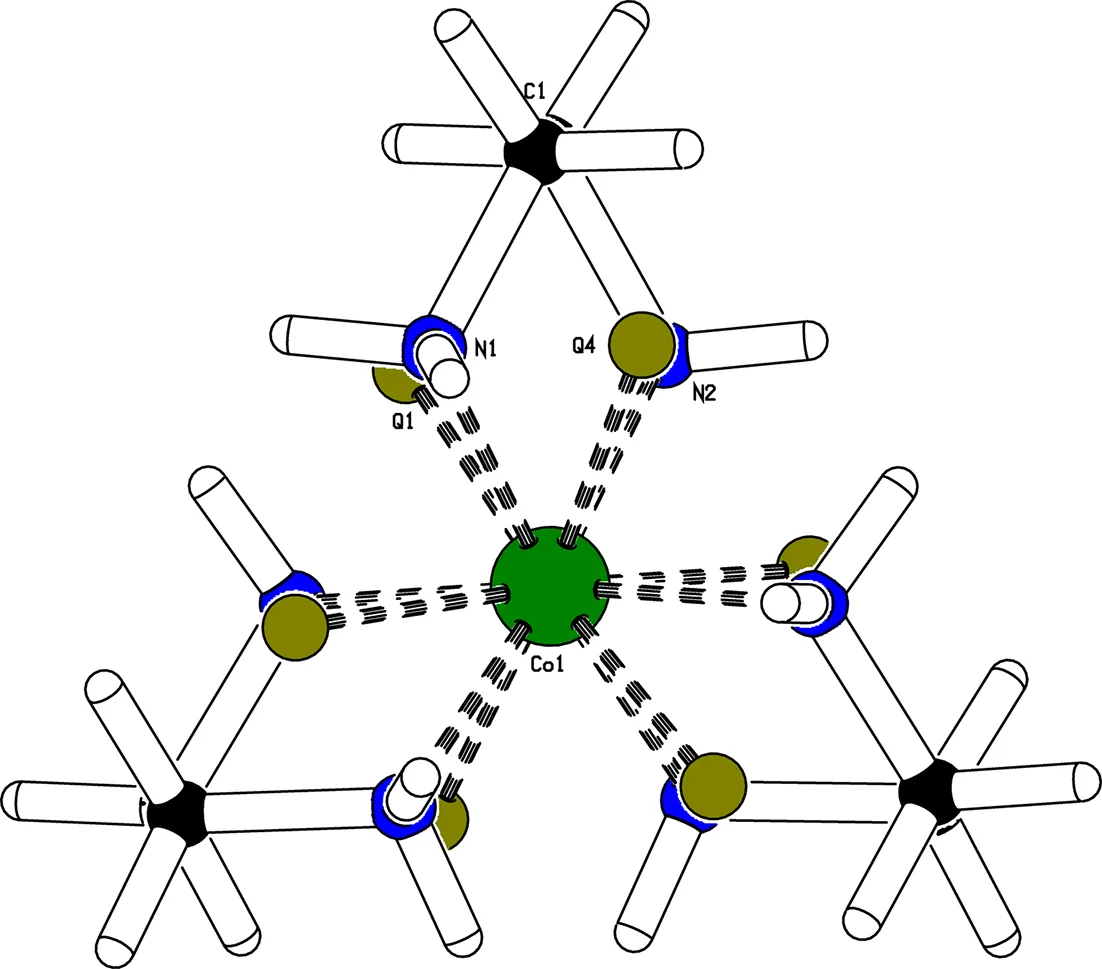
Shown in yellow–greenish are the largest difference electron-density peaks in the cation region of the preliminary refined structure CIJVEA in the space group *P*
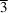
*c*1. They indicate a twofold axial twinning parallel to the threefold symmetry axis of the cation and lattice.

**Figure 4:**
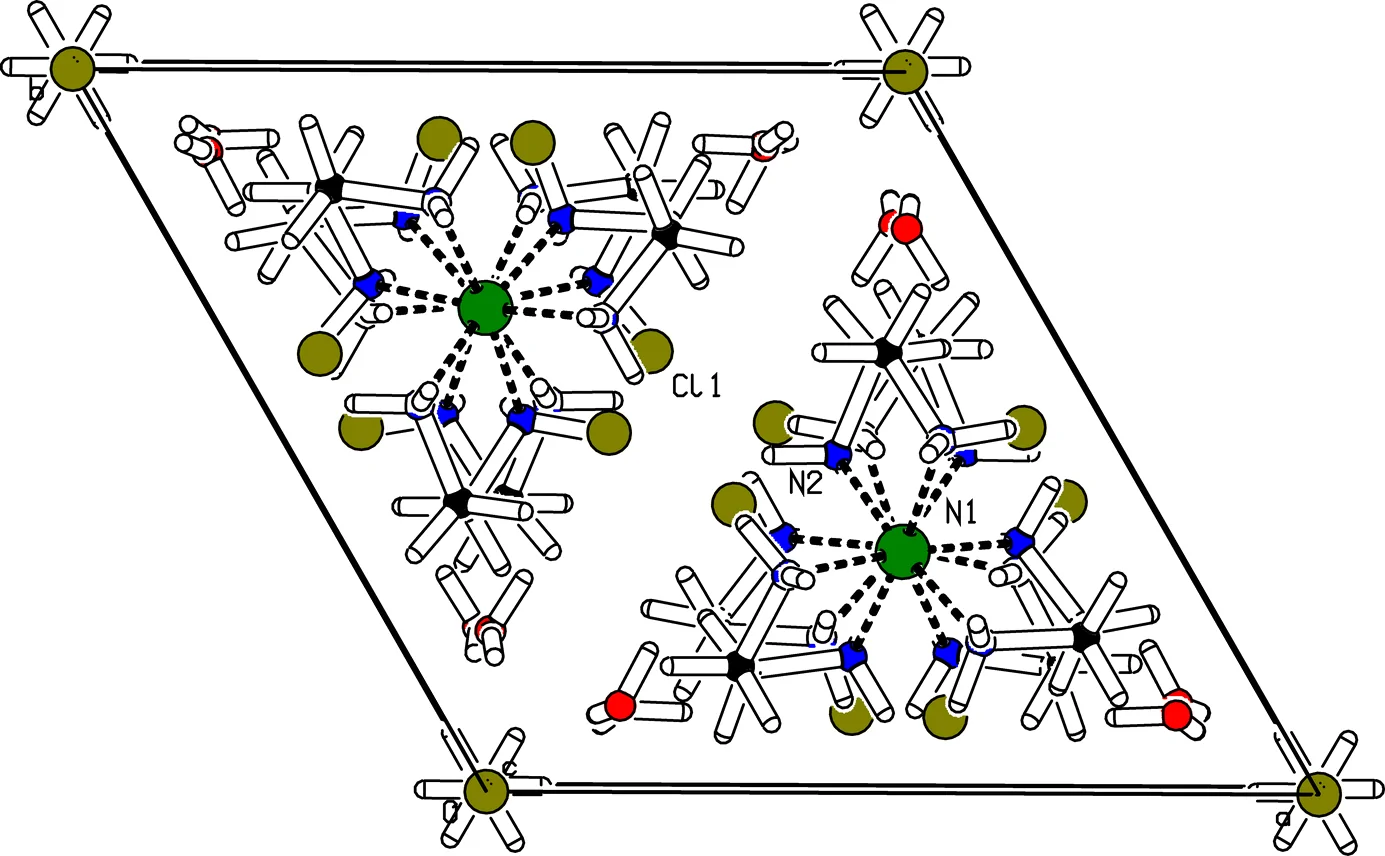
Crystal structure of CIJVEA in the space group *P*
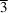
*c*1, twinned by 180° rotation about [001], colinear with the threefold axis through the central Co^III^ atom of the cation.

**Figure 5:**
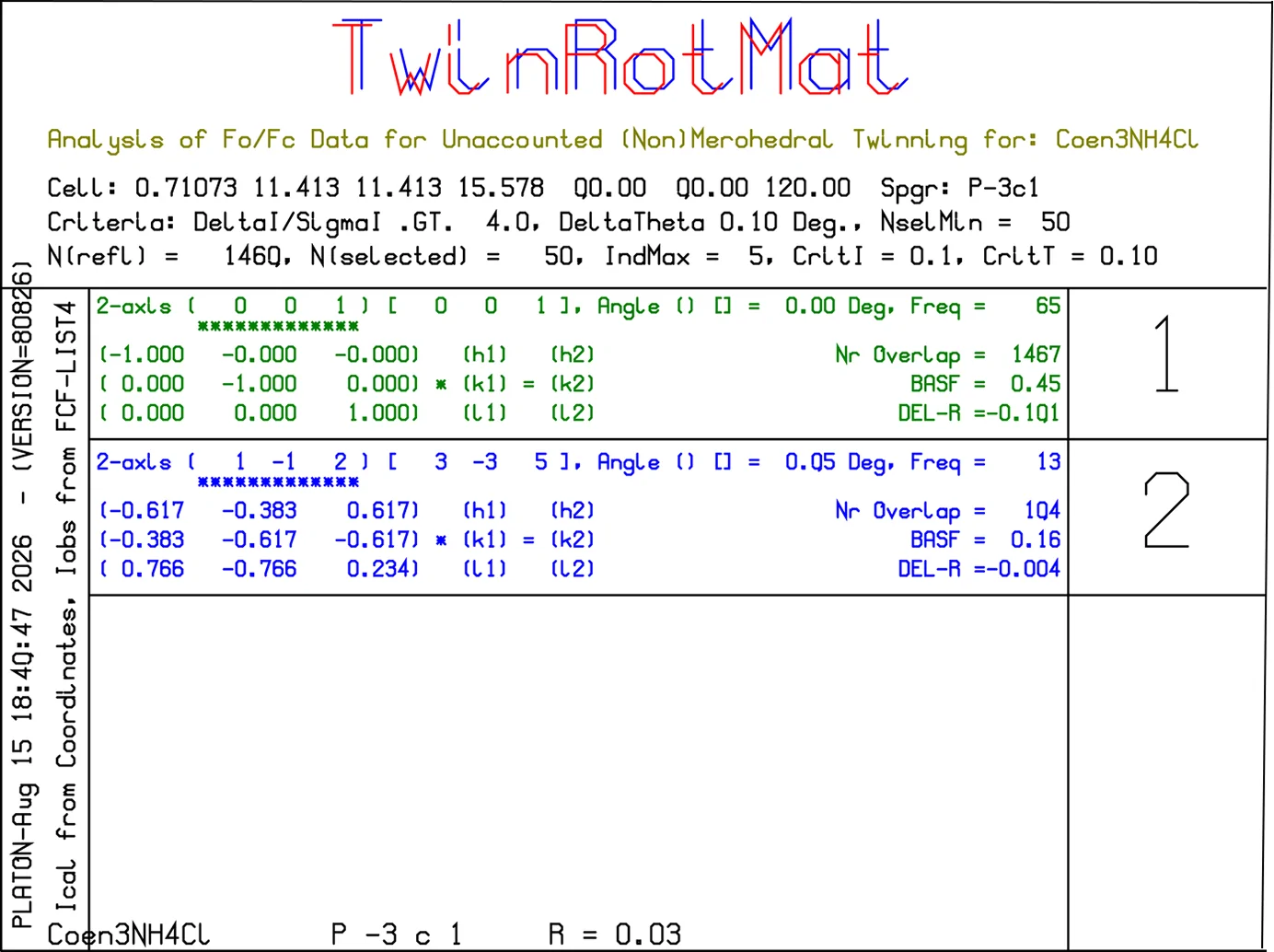
*TwinRotMat* report for the merohedrally twinned CIJVEA structure showing twofold rotational twinning about [001] with the expected *R*1 value drop when included in the refinement. The second proposed twinning option appears to have no effect on the *R*1 value.

**Figure 6:**
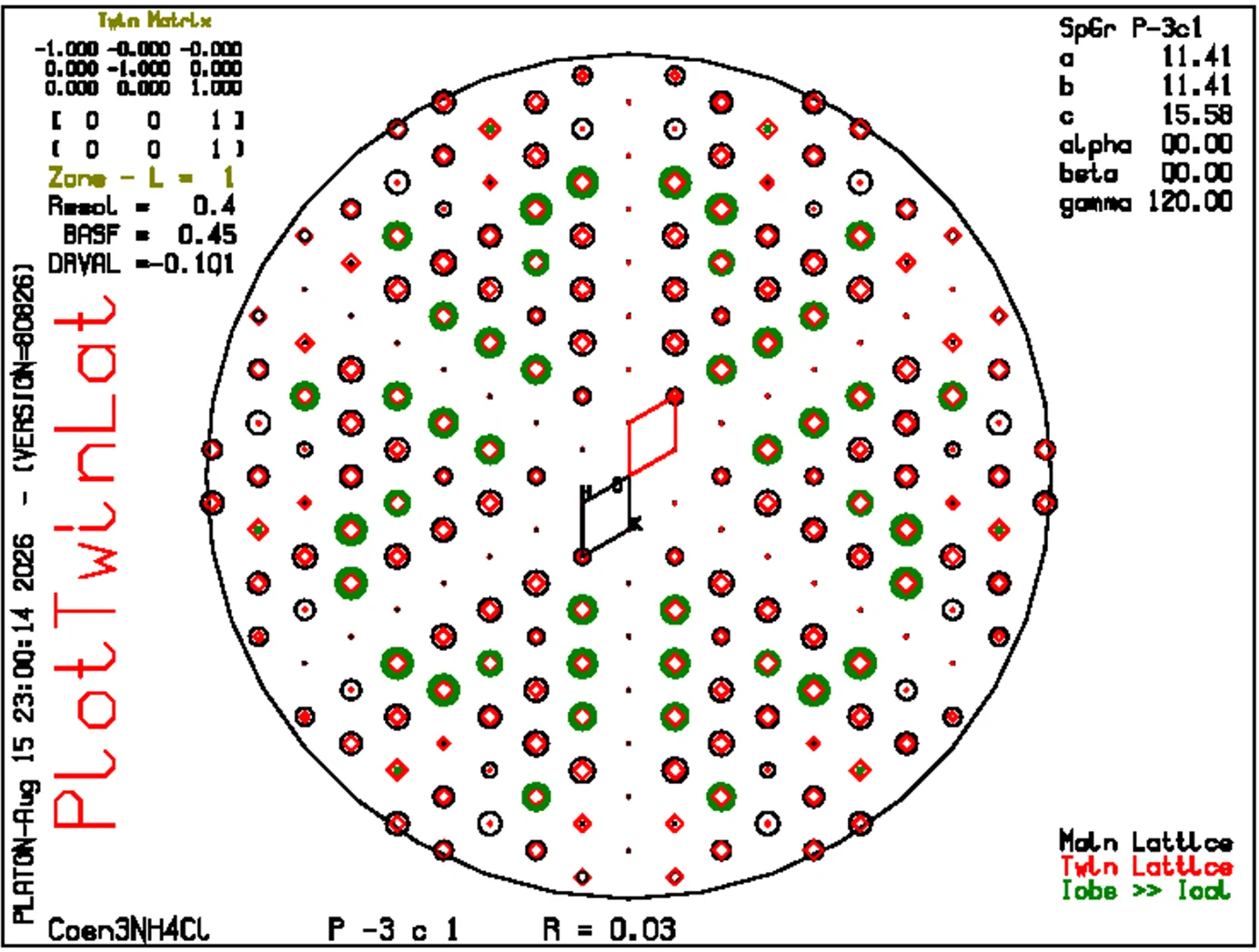
Display of the *l* = 1 zone of the reciprocal lattice showing the complete overlap of the twin related lattices for CIJVEA. Reflections with large *F*_o_^2^ >> *F*_c_^2^ values are displayed with green circles.

**Figure 7:**
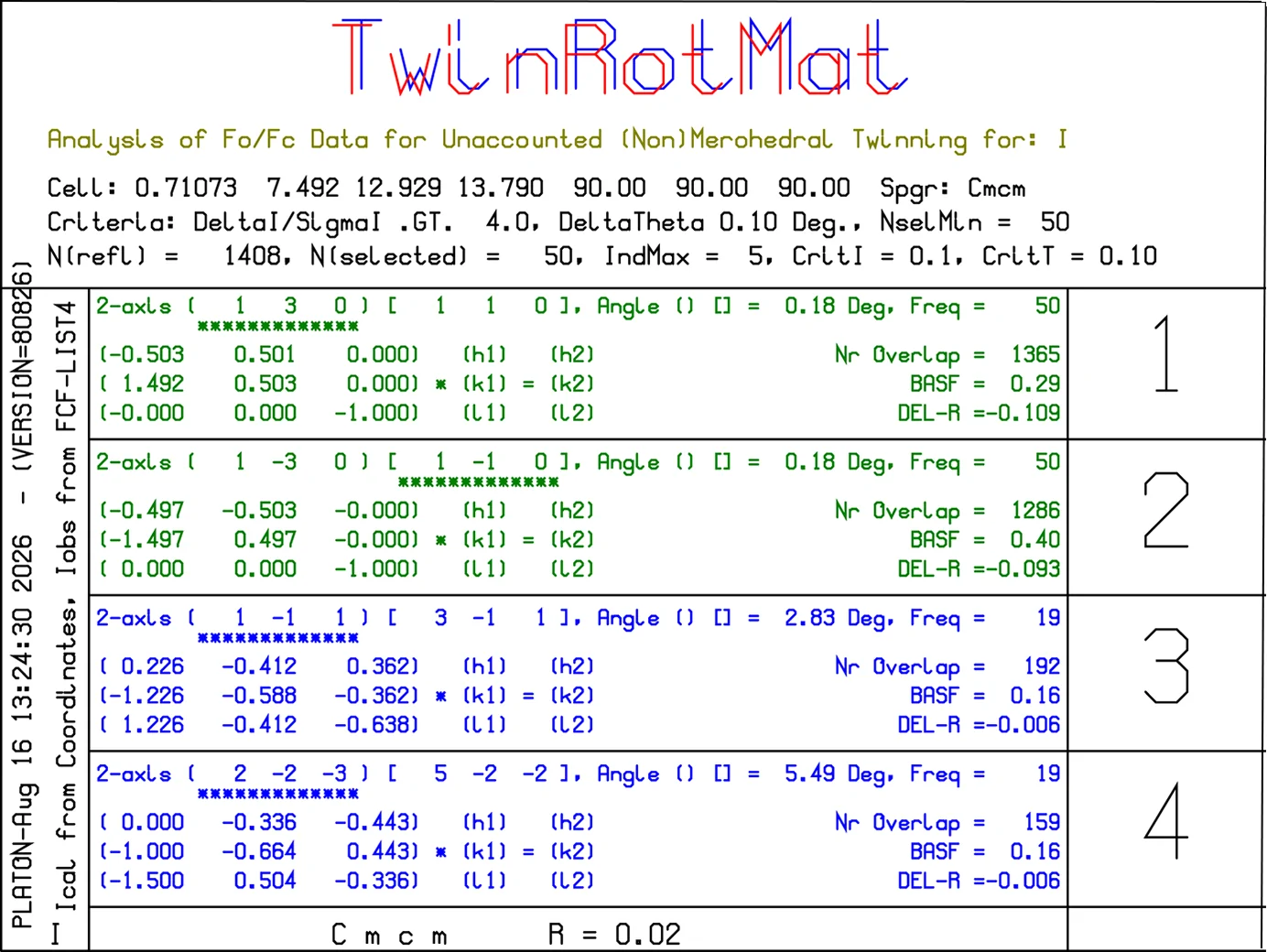
*TwinRotMat* report for the pseudomerohedrally twinned WUXXEU structure. Two twofold twinning axes, [110] and [1
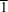
0], are indicated.

**Figure 8:**
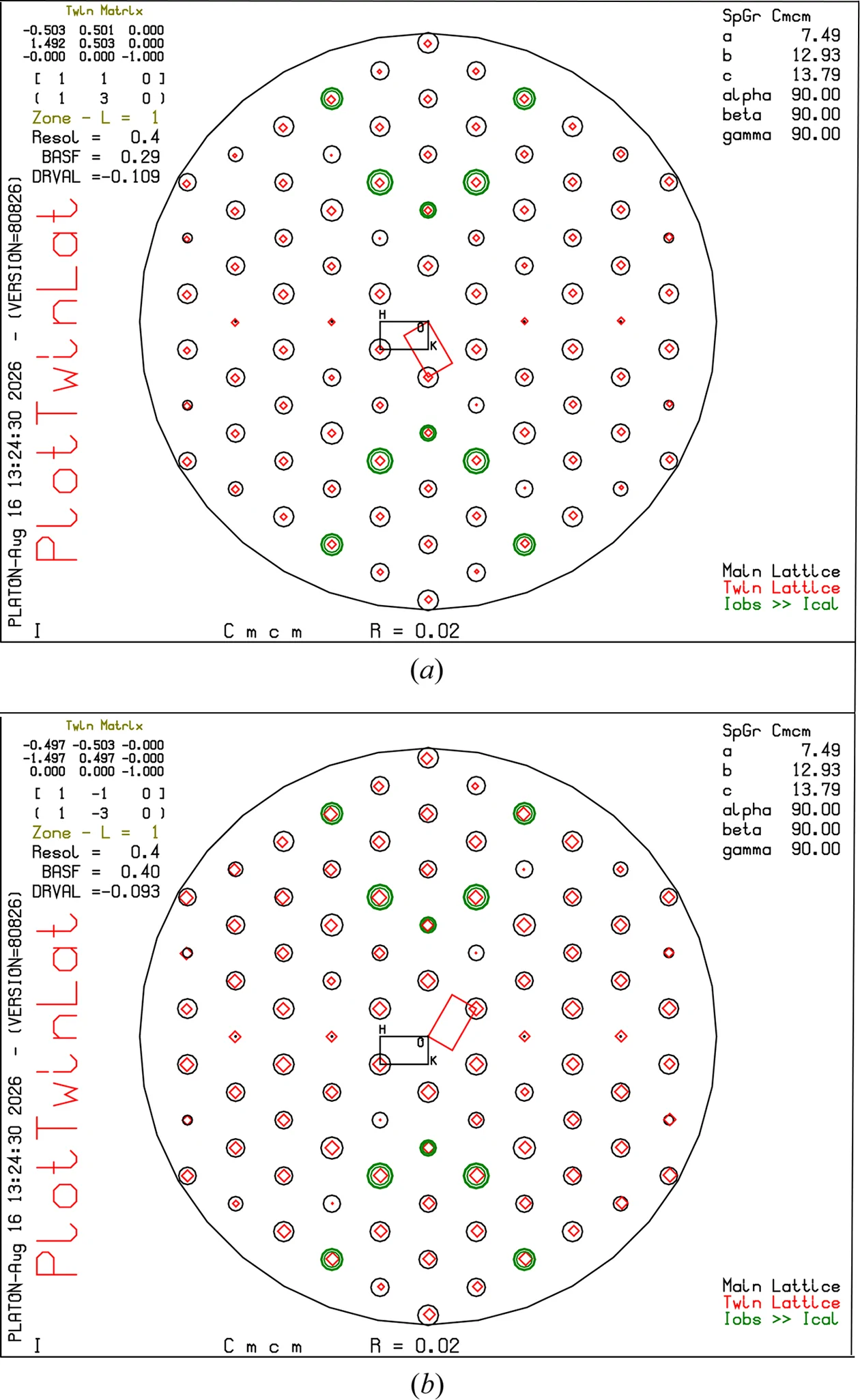
Display of the approximate reciprocal lattice overlap for the *l* = 1 zone for (*a*) the twofold twin operation [110] and (*b*) the twofold twin operation about [1
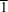
0] for WUXXEU

**Figure 9:**
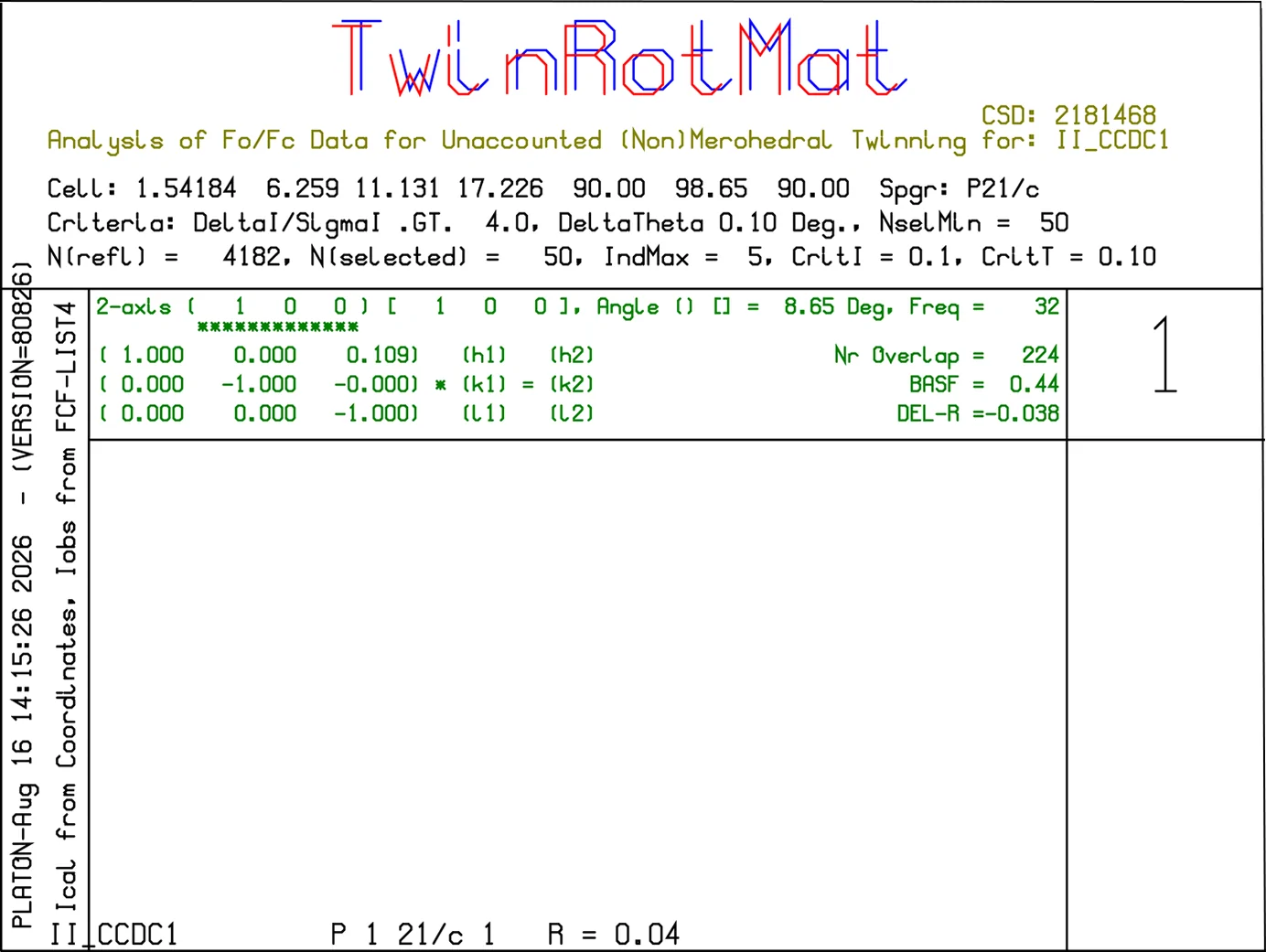
*TwinRotMat* report for LUMKIP01. The structure is non-merohedrally twinned with a 180° rotation about a*.

**Figure 10:**
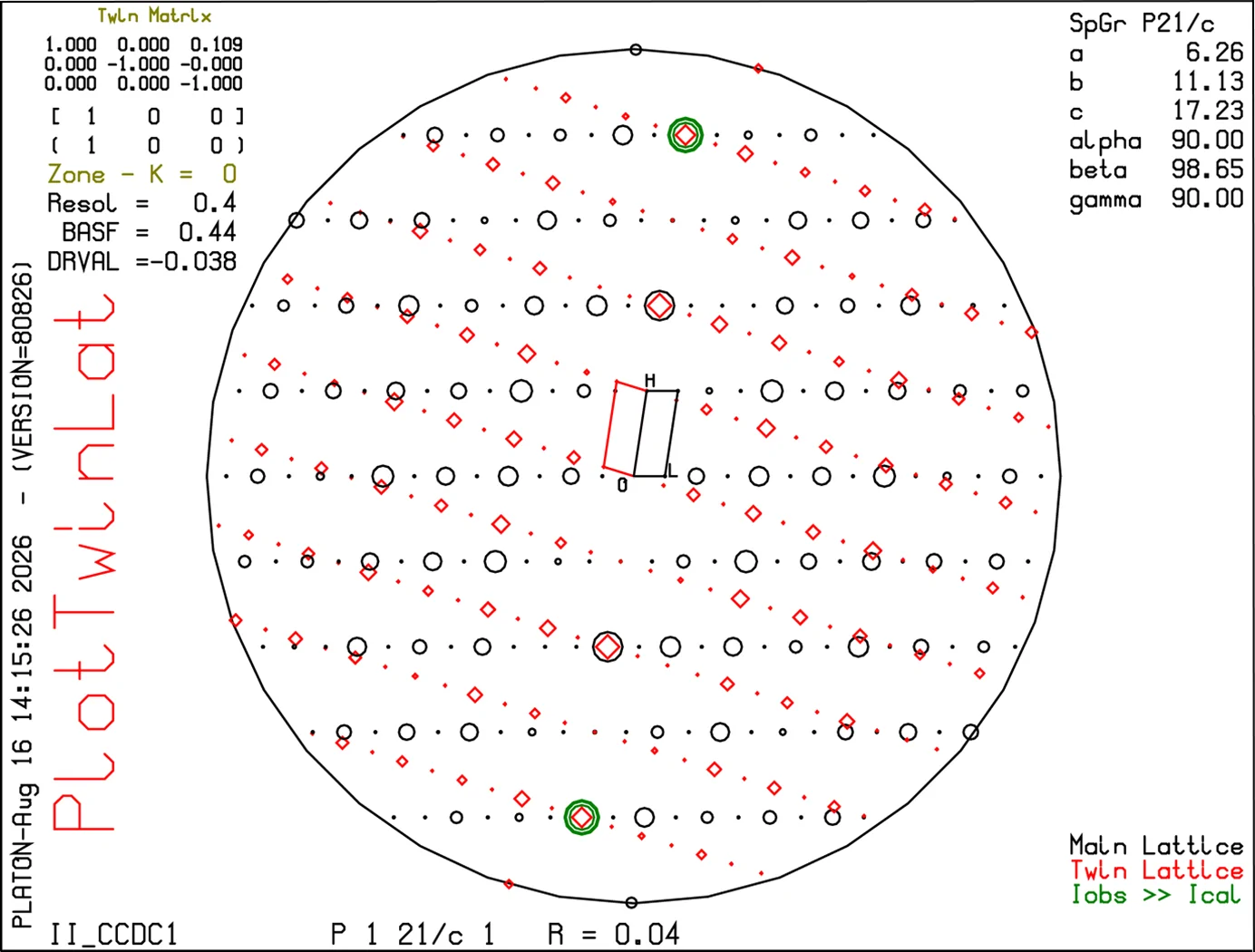
Display of the *k* = 0 reciprocal lattice zone of the non-merohedrally twinned structure of LUMKIP01.
